# Myocardial Injection of Apelin-Overexpressing Bone Marrow Cells Improves Cardiac Repair via Upregulation of Sirt3 after Myocardial Infarction

**DOI:** 10.1371/journal.pone.0071041

**Published:** 2013-09-06

**Authors:** Lanfang Li, Heng Zeng, Xuwei Hou, Xiaochen He, Jian-Xiong Chen

**Affiliations:** Department of Pharmacology and Toxicology, University of Mississippi Medical Center, Jackson, Mississippi, United States of America; Indiana University, United States of America

## Abstract

Our previous study shows that treatment with apelin increases bone marrow cells (BMCs) recruitment and promotes cardiac repair after myocardial infarction (MI). The objective of this study was to investigate whether overexpression of apelin in BMCs improved cell therapy and accelerated cardiac repair and functional recovery in post-MI mice. Mouse myocardial infarction was achieved by coronary artery ligation and BMCs overexpressing apelin (apelin-BMCs) or GFP (GFP-BMCs) were injected into ischemic area immediately after surgery. *In vitro*, exposure of cultured BMCs to apelin led to a gradual increase in SDF-1á and CXCR4 expression. Intramyocardial delivery of apelin-BMCs in post-MI mice resulted in a significant increase number of APJ^+^/c-kit^+^/Sca1^+^ cells in the injected area compared to GFP-BMCs treated post-MI mice. Treatment with apelin-BMCs increased expression of VEGF, Ang-1 and Tie-2 in post-MI mice. Apelin-BMCs treatment also significantly increased angiogenesis and attenuated cardiac fibrosis formation in post-MI mice. Most importantly, treatment with apelin-BMCs significantly improved left ventricular (LV) systolic function in post-MI mice. Mechanistically, Apelin-BMCs treatment led to a significant increase in Sirtuin3 (Sirt3) expression and reduction of reactive oxygen species (ROS) formation. Treatment of cultured BMCs with apelin also increased Notch3 expression and Akt phosphorylation. Apelin treatment further attenuated stress-induced apoptosis whereas knockout of Sirt3 abolished anti-apoptotic effect of apelin in cultured BMCs. Moreover, knockout of Sirt3 significantly attenuated apelin-BMCs-induced VEGF expression and angiogenesis in post-MI mice. Knockout of Sirt3 further blunted apelin-BMCs-mediated improvement of cardiac repair and systolic functional recovery in post-MI mice. These data suggest that apelin improves BMCs therapy on cardiac repair and systolic function in post-MI mice. Upregulation of Sirt3 may contribute to the protective effect of apelin-BMCs therapy.

## Introduction

Both experimental and clinical studies show that treatment of myocardial ischemia with bone marrow derived cells (BMCs) reduces infarct size and improves cardiac function [1], [2]. BMCs have been identified as promising candidate for use as cell based therapy for cardiac regeneration and repair after myocardial infarction (MI) [3], [4]. However, one of the significant problems with BMCs therapy is cell survival. Myocardial ischemia creates a hostile environment due to locally increased reactive oxygen species (ROS) formation inducing cell apoptosis. Accumulative evidence reveals that survival of the injected BMCs is very low, especially in post-MI hearts. To circumvent this shortcoming of cell based therapy in ischemic hearts, one of the therapeutic approaches is overexpression of mesenchymal stem cells with pro-survival factors. Overexpression of mesenchymal stem cells with Akt or glycogen synthase kinase-3 (GSK-3) in *ex vivo* increases the survival of mesenchymal stem cells after transplantation and significantly improves cardiac function in post-MI mice [5], [6]. These studies suggest that a combination of BMCs and gene therapy may be attractive strategy to boost BMCs-based cell therapies for heart failure.

Apelin is an endogenous ligand for the angiotensin-like 1 receptor (APJ) and has beneficial effects against myocardial ischemia/reperfusion injury [7]–[10]. Transplantation of BMCs led to a significant increase in apelin level and improvement of cardiac function in patients with severe heart failure [11]. In contrast, loss of apelin and APJ function impairs differentiations of endothelial, hematopoietic and cardiac progenitor cell [12], [13]. Treatment of BMCs with apelin also attenuates starvation-induced bone marrow mesenchymal cells apoptosis via activation of PI3k/Akt pathway [14]. Our recent study demonstrated that treatment with apelin led to a significant increase in homing of BMC derived vascular progenitor cells and improvement of cardiac function in post-MI mice. This was accompanied by a significant upregulation of angiogenic growth factors such as VEGF and Notch3, and improvement of angiogenesis [15]. Sirtuin 3 (Sirt3) belongs to a highly conserved family of protein deacetylases and its activity is closely associated with the prolong lifespan of human [16], [17]. Sirt3 has been shown to protect cardiomyocytes from oxidative stress-mediated cell death [18], [19]. Overexpression of Sirt3 blocked cardiac hypertrophy by suppressing ROS formation. In contrast, knockout of Sirt3 promoted angiotensin II-induced cardiac hypertrophy in mice [19], [20]. In this study, we tested our hypothesis that overexpression of apelin in BMCs enhances cardiac repair and functional recovery by a mechanism involving upregulation of Sirt3 and angiogenesis in post-MI mice.

Using BMCs isolated from wild type (WT) mice and Sirt3 knockout (Sirt3KO) mice, the present study was: (i) to determine whether overexpression of apelin in WT-BMCs or Sirt3KO-BMCs improved cell therapy and enhanced cardiac repair; and (ii) to examine the potential molecular mechanism by which intramyocardial injection of apelin-BMCs improved cardiac repair in post-MI mice.

## Methods

### Ethics Statement

All procedures conformed to the Institute for Laboratory Animal Research Guide for the Care and Use of Laboratory Animals and were approved by the University of Mississippi Medical Center Animal Care and Use Committee (Protocol ID: 1280). The investigation conforms to the Guide for the Care and Use of Laboratory Animals published by the US National Institutes of Health (NIH Publication No. 85–23, revised 1996).

### Bone marrow cells (BMCs) culture

To assess the role of apelin on BMCs activity of post-MI mice *in vivo*, the experimental mice received intraperitoneal (i.p.) apelin (1 mg/kg/d, Cayman Chemical, MI) daily for 14 days after myocardial ischemia. At 24 hours and 14 days of myocardial ischemia, BM–derived mononuclear cells were obtained by flushing the tibias and femurs with 10% FBS EGM. Immediately after isolation, 10^5^ BM–derived mononuclear cells were plated into 6-well culture plates. After 4 days of culture, the nonadherent cells were removed and the adherent cells were washed three times with phosphate-buffered saline solution (PBS). BMC isolated from post-MI mice were cultured and harvested for the western blot analysis [15], [21].

### BM colony-forming cell assay

BM–derived mononuclear cells were isolated from sham control, post-MI 14 days, apelin treated sham control and apelin treated post-MI mice. BMCs (10^5^ cells per dish) were then seeded in 2% methylcellulose medium. After 7 days of incubation, BMCs colony formation and colony number were scored under phase-contrast microscopy [22].

### Overexpression of apelin in donor BM

**Figure 1:**
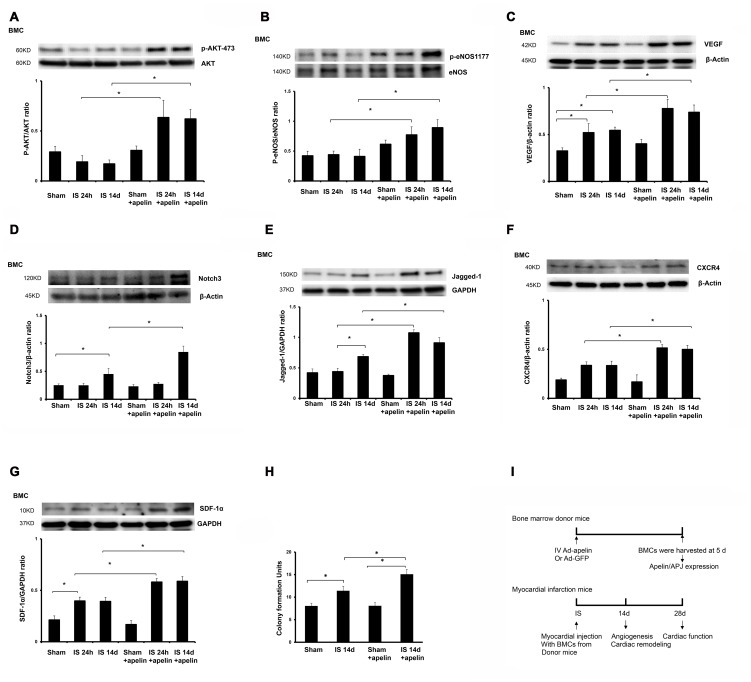
Apelin treatment increases expression of pro-survival and pro-angiogenic growth factors in BMCs. **A and B**. Representative phosphorylation levels of Akt-(473) and eNOS-(1177) in cultured BMCs of post-MI mice. The phosphorylation of Akt-(473) and eNOS-(1177) was significantly enhanced in cultured BMCs from apelin-treated post-MI mice (n = 4 mice, *p<0.05). **C**. Expression of VEGF in cultured BMCs of post-MI mice. VEGF expression in cultured BMCs was significantly upregulated in apelin treated post-MI mice compared to saline-treated mice at 24 hour and 14 days of post-MI (n = 4 mice, *p<0.05). **D and E**. Expression of Notch3 and Jagged1 in cultured BMCs of post-MI mice. Notch3 expression in cultured BMCs was significantly increased in control post-MI mice compared with sham control mice at 14 days. Treatment with apelin led to a significant increase in Notch3 expression in cultured BMCs of post-MI mice compared to control post-MI mice at 14 days (n = 4 mice, *p<0.05). Jagged1 expression in cultured BMCs was significantly increased in control post-MI mice compared with sham control mice at 14 days. Treatment with apelin resulted in a significant increase in Jagged1 expression compared to control post-MI mice (n = 4 mice, *p<0.05). **F and G**. CXCR-4 and SDF-1α expression in cultured BMCs of post-MI mice. The expression of CXCR-4 and SDF-1α was significantly upregulated in cultured BMCs of the apelin treated post-MI mice compared to saline-treated post-MI mice at 24 hour and 14 days (n = 4 mice, *p<0.05). **H**. BMC colony formation units. Apelin treatment led to a significantly increase in colony formation in cultured BMCs compared to saline-treated mice at 14 days of post-MI (n = 6 mice, *p<0.05). I. Schematic diagram of apelin-BMCs treatment and experimental endpoints *in vivo*.

The donor mice (WT mice or Sirt3 knockout mice, 12 weeks old) received intravenous tail vein injection of Ad-apelin (1×10^9^ PFU) or Ad-GFP (1×10^9^ PFU) [21], [23]. At day 5, WT mice or Sirt3KO mice were sacrificed by cervical dislocation under anesthesia with isoflurane. Mice tibias and femurs were harvested and flushed with PBS to retrieve BM–derived mononuclear cells (Figure 1–I). Overexpression of apelin in the donor BM cells was verified by western blot analysis.

### Surgical procedures

Male WT mice (10 weeks old) were anesthetized with ketamine (100 mg/kg) plus xylazine (15 mg/kg), intubated, and artificially ventilated with room air. Adequate anesthesia was monitored by toe pinch. Myocardial infarction was achieved by ligation of the left anterior descending coronary artery (LAD). Sham controls underwent surgery without the LAD [15], [21], [23]. After induction of myocardial ischemia (IS), mice were immediately intramyocardial injected with donor WT or Sirt3KO BM–derived mononuclear cells (1×10^6^ cells) either overexpressing GFP or apelin (Figure 1–I).

### Western analysis of Sirt3, VEGF, Ang-1, Tie-2, eNOS, Akt, Jagged1, Notch3, CXCR-4, SDF-1α, p47^phox^, gp91^phox^, beclin-1 and LC3-I/II expression

The hearts or cultured BMCs were harvested and homogenized in lysis buffer for Western blot analysis. The membranes were immunoblotted with Ang-1 (Sigma Co, MO), beclin-1, LC3-I/II, Sirt3, Tie-2, Akt and eNOS, (Cell signaling, MA), p47^phox^ and gp91^phox^, APJ, VEGF, Jagged1, Notch3, CXCR-4 and SDF-1α (1∶1000, Santa Cruz, CA), and apelin (abcam, MA) antibodies. The densitometric analysis was carried out using image acquisition and analysis software (TINA 2.0).

### Analysis of APJ^+^/Sca1^+^/c-kit^+^ cells in the heart

Heart tissue sections (8 µm) from injected area of ischemia were incubated with APJ, Sca1 and c-kit (1∶200 Santa Cruz, CA) antibodies overnight. Sca1 and c-kit were visualized using FITC labeled goat anti-mouse IgG antibodies; APJ was visualized with Fluorolink™ Cy™3 labeled goat anti-mouse IgG antibodies. Myocardial APJ^+^/Sca1^+^ and c-kit^+^/APJ^+^ cells in the injected area were assessed by counting the number of positive cells per 100 nuclei [15], [24].

### Analysis of myocardial capillary and arteriole densities

Eight-micrometer heart tissue sections were cut and incubated with fluorescerin-labeled Isolectin B4 (1∶200; IB4, Molecular Probe, Invitrogen, OR) and Cy3-conjugated anti-α smooth muscle actin (SMA) (1∶100; Sigma). The number of capillaries (IB4-positive EC) was counted and expressed as capillary density per square millimeter (mm^2^) of tissue. Myocardial arteriole density was measured using image analysis software (Image J, NIH, MD) [15], [23].

### Myocardial apoptosis and ROS formation

Heart tissue sections were stained with transferase deoxyuridine nick end labeling (TUNEL) following the manufacturer's instructions (Promega, WI). Apoptosis was indexed by counting TUNEL positive cells per 100 nuclei in the infarcted tissue [15], [21]. ROS formation was measured and quantified by staining with DHE as previously described [25], [26].

### Cardiac function

Experimental mice were anesthetized with ketamine (100 mg/kg) plus xylazine (15 mg/kg), intubated and artificially ventilated with room air. A 1.4-Fr pressure–conductance catheter (SPR-839, Millar Instrument, TX) was inserted into the left ventricle (LV) to record baseline cardiac hemodynamics of the hearts [15], [24].

### Heart weight to body weight ratio (HW/BW) and fibrosis

Cardiac hypertrophy was assessed by measuring heart-to-body weight ratio at 14 days of post-MI. Cardiac β-myosin heavy chain (β-MHC) and atrial natriuretic peptide (ANP) expression were examined by western blot analysis. Cardiac fibrosis was stained with Masson's trichrome (MT, Sigma, MO) and quantified by measuring the blue fibrotic area [23].

### Statistical analysis

The results are expressed as the mean ±SD. Statistical analysis was performed using one way ANOVA followed by post hoc multiple comparisons test. Significance was set at *P*<0.05.

## Results

### The expression of angiogenic growth factors is increased in BMCs of apelin treated post-MI mice

Our recent study demonstrates that treatment of ischemic hearts with apelin increased the recruitment of BM derived progenitor cell into ischemia area [15]. However, the effect of apelin on the numbers and functional activity of BMCs in post-MI mice remains unknown. To test whether apelin regulated BMCs function, the expression of pro-survival and pro-angiogenic growth factors were examined in cultured BMCs isolated from post-MI mice. The phosphorylation of Akt-473 and eNOS-1177 was significantly increased in cultured BMCs of apelin-treated post-MI mice. Similarly, the levels of VEGF, CXCR4, SDF-1á, Jagged1 and Notch3 expression were significantly upregulated in cultured BMCs of apelin-treated post-MI mice (**Fig. 1 A–G**). Moreover, the colony formation was significantly increased in BMCs of apelin-treated post-MI mice (**Fig. 1H**).

### Apelin-BMCs treatment enhances angiogenic growth factors expression and angiogenesis in hearts of post-MI mice

**Figure 2:**
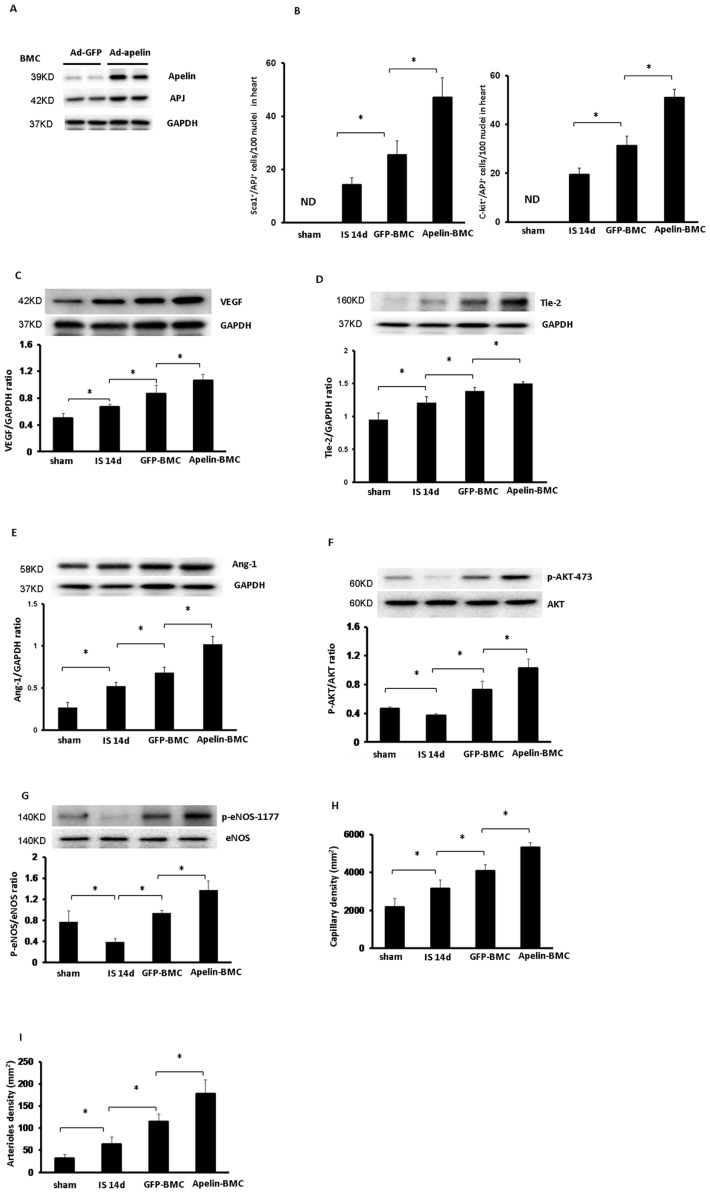
Apelin-BMC treatment augments expression of angiogenic growth factors and promotes myocardial angiogenesis in post-MI mice. **A**. Western blot analysis revealing that systemic delivery of Ad-apelin in WT mice for 5 days upregulated apelin and APJ expression in the BMCs. n = 2 mice. **B**. Quantitative analysis of APJ^+^/Sca1^+^/c-kit^+^ cells demonstrating that Apelin-BMCs treated post-MI mice had a significant higher number of APJ^+^/Sca1^+^/c-kit^+^ cells in the injected area compared to GFP-BMCs treated mice. All data represent mean ± SD; n = 5, *p<0.05. ND = not detected. **C to G**. Treatment with GFP-BMCs significantly increased VEGF, Tie2 and Ang-1 expression in post-MI mice compared to that of control post-MI mice. The expression of VEGF, Tie2 and Ang-1 was significantly upregulated in the apelin-BMCs treated mice compared to GFP-BMCs treatment (n = 5 mice, *p<0.05). GFP-BMCs treatment significantly increased phosphorylation of Akt-473 and eNOS-1177 in post-MI mice compared to that of control post-MI mice. The phosphorylation of Akt-473 and eNOS-1177 was significantly increased in the apelin-BMCs treated mice compared to GFP-BMCs treatment. n = 5 mice; *p<0.05. **H**. GFP-BMCs treatment significantly increased capillary formation compared to control post-MI mice. Treatment with apelin-BMCs led to a further increase in myocardial capillary density compared to GFP-BMCs treatment. n = 5 mice; *p<0.05. **I**. GFP-BMCs treatment significantly increased myocardial arteriole density compared to control post-MI mice at 14 days. Treatment with apelin-BMCs significantly increased myocardial arteriole density compared to GFP-BMCs treatment. n = 5 mice; *p<0.05.

Systemic delivery of Ad-apelin in donor mice for 5 days increased apelin and APJ expression in BMCs as compared to Ad-GFP treated mouse BMCs (**Fig. 2A**). The number of APJ^+^/c-kit^+^/Sca1^+^ cells in the injected area was evaluated after BMCs intramyocardial injection. As shown in **Fig. 2 B**, apelin-BMCs treatment had a significant higher number of APJ^+^/c-kit^+^/Sca1^+^ cells in comparison with GFP-BMCs treatment. Next, we investigated whether intramyocardial injection with apelin-overexpressing BMCs affected angiogenic growth factors expression and angiogenesis in the ischemic border zone of post-MI mice. Treatment with GFP-BMCs significantly increased expression of VEGF, Ang-1 and Tie-2 as well as phosphorylation of Akt-473 and eNOS-1177. The expression of VEGF, Ang-1 and Tie-2 and phosphorylation of Akt-473 and eNOS-1177 were significantly higher in the border zone of apelin-BMCs treated mice compared to GFP-BMCs treated mice (**Fig. 2 C–G**). Apelin-BMCs treatment further augmented myocardial angiogenesis compared to GFP-BMCs treatment (**Fig. 2 H and I**).

### Apelin-BMCs treatment attenuates cardiac hypertrophy and fibrosis

**Figure 3:**
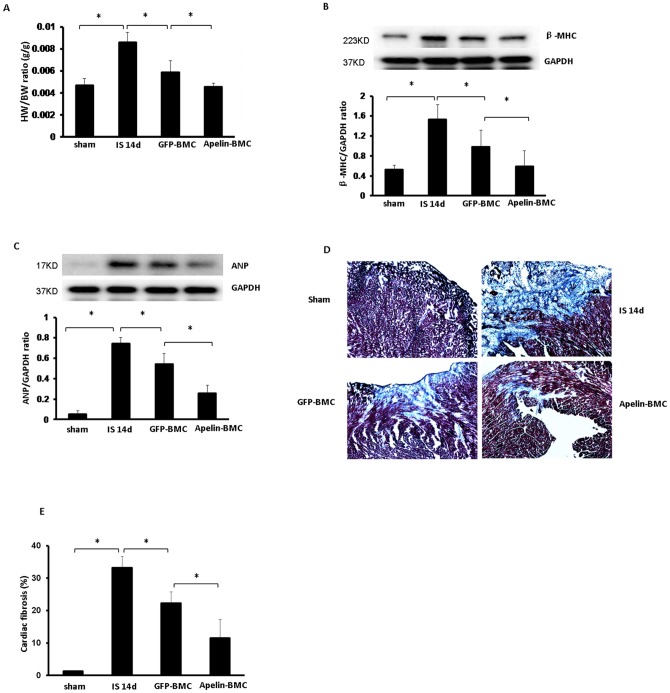
Apelin-BMCs treatment inhibits cardiac hypertrophy and fibrosis formation. **A**. Treatment with GFP-BMCs significantly reduced HW/BW ratio compared to saline treatment. Treatment with apelin-BMCs led to a further significant reduction of HW/BW ratio compared to GFP-BMCs treated group. n = 4–6 mice, *p<0.05. **B and C**. GFP-BMCs treatment significantly decreased heart β-MHC and ANP expression compared to saline treatment. Treatment with apelin-BMCs led to a further decrease in β-MHC and ANP expression compared to GFP-BMCs group. n = 4 mice, *p<0.05. **D and E**. Representative images of cardiac fibrosis in the infarction zone and quantitative analysis of fibrotic area in mice (Masson's trichrome). The area of cardiac fibrosis (blue) in post-MI mice was increased significantly at 14 days of MI. GFP-BMCs treatment significantly reduced the area of cardiac fibrosis compared to those treated with saline. Apelin-BMCs treatment significantly decreased cardiac fibrosis area compared to GFP-BMCs treated group. n = 5 mice; *p<0.05.

The HW/BW ratio and levels of β-MHC and ANP expression were dramatically reduced in GFP-BMCs treated post-MI mice compared to saline treated post-MI mice (**Fig. 3 A–C**). Treatment with Apelin-BMCs further reduced cardiac hypertrophy compared to GFP-BMCs treatment (**Fig. 3 A–C**). Moreover, Apelin-BMCs treatment had a greater inhibitory effect on fibrosis formation in post-MI mice (**Fig. 3 D&E**).

### Apelin-BMCs treatment increases Sirt3 and attenuates ROS formation in the heart of post-MI mice

**Figure 4:**
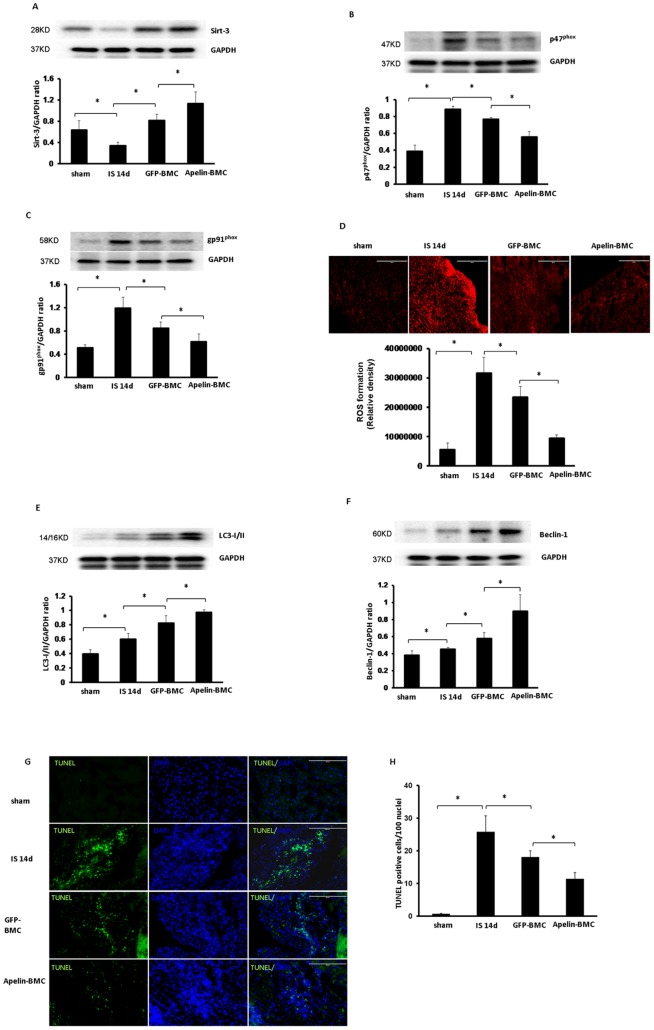
Apelin-BMCs treatment upregulates Sirt3 expression and attenuates ROS formation. **A**. Treatment with GFP-BMCs significantly increased Sirt-3 expression compared to mice treated with saline. Treatment with apelin-BMCs led to a further significant increase in Sirt-3 expression compared to GFP-BMCs treatment. n = 4 mice, *p<0.05. **B and C**. The expression p47^phox^ and gp91^phox^ was significantly increased at 14 days of post-MI mice compared to sham control mice. GFP-BMCs treatment significantly reduced p47^phox^ and gp91^phox^ expression compared to post-MI mice treated with saline. Apelin-BMCs treatment led to a significant reduction in p47^phox^ and gp91^phox^ expression compared to GFP-BMCs treatment. n = 4 mice, *p<0.05. **D**. DHE staining and quantitative analysis showing that ROS formation was significantly increased in post-MI mouse hearts at 14 days compared to sham control. Treatment with GFP-BMCs significantly decreased ROS formation compared to that of control post-MI mice. ROS formation was significantly reduced in the apelin-BMCs treated mice compared to GFP-BMCs treated mice. n = 5 mice; *p<0.05. **E and F**. LC3-I/II and Beclin-1 expression was significantly increased in the mouse hearts of post-MI compared to sham control. GFP-BMCs treatment significantly increased LC3-I/II and Beclin-1 expression compared to that of control post-MI mice. The expression of LC3-I/II and Beclin-1 was significantly increased in the apelin-BMCs treated mice compared to GFP-BMCs treated mice. n = 4 mice; *p<0.05. **G and H**. Apoptotic cells in the infarcted area of the left ventricle were identified by TUNEL staining (green, 10×) and total nuclei by DAPI counterstaining (blue, 10×). GFP-BMCs significantly decreased TUNEL^+^ nuclei in post-MI mice compared to that of control post-MI mice. TUNEL^+^ nuclei were significantly decreased in the apelin-BMCs treated mice compared to GFP-BMCs treated mice. n = 6 mice; *p<0.05.

Treatment with GFP-BMCs led to a significant increase in Sirt3 expression and reduction of NADPH oxidase subunits p47^phox^ and gp91^phox^ expression in post-MI mice (**Fig. 4A–C**). Treatment with GFP-BMCs also significantly reduced ROS formation in post-MI mice (**Fig. 4D**). Apelin-BMCs treatment further augmented Sirt3 expression together with greater inhibition on p47^phox^ and gp91^phox^ expression. The levels of intracellular ROS was significantly reduced in apelin-BMCs treated hearts compared to GFP-BMCs treated hearts (**Fig. 4 A–D**).

### Apelin-BMCs treatment increases autophagy gene expression and reduces myocardial apoptosis

Treatment of post-MI mice with GFP-BMCs led to a significant increase in autophagy gene LC3-I/II and beclin-1 expression (**Fig. 4 E and F**). This was accompanied by a dramatic decrease in myocardial apoptosis in post-MI mice. Apelin-BMCs treatment further enhanced LC3-I/II and beclin-1 expression compared to GFP-BMCs treatment. Apelin-BMCs treatment also significantly boosted BMCs-mediated suppression on myocardial apoptosis (**Fig. 4 G&H**).

### Apelin-BMCs treatment improves systolic function in post-MI mice

**Figure 5:**
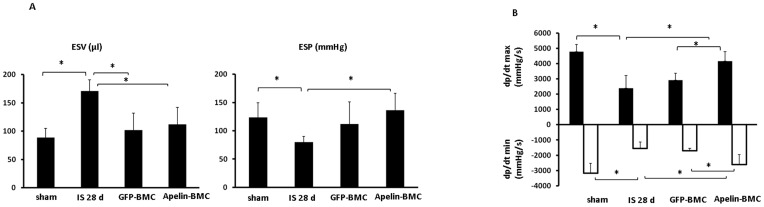
Treatment with apelin-BMCs improves heart systolic function at 28 days of post-MI mice. **A**. The end-systolic volume (ESV) was significantly increased whereas the end-systolic pressure (ESP) was decreased in post-MI mice. GFP-BMCs and apelin-BMCs treatment significantly decreased ESV compared to saline-treated mice. Treatment with apelin-BMCs, but not GFP-BMCs, led to a significant increase in ESP compared to saline-treated post-MI mice. n = 5–8 mice, *p<0.05. **B**. Maximum +dP/dt pressure was increased whereas minimum −dP/dt pressure was decreased in the apelin-BMCs treated mice compared to saline-treated and GFP-BMCs-treated mice. n = 5–8 mice, *p<0.05.

To further investigate whether treatment with apelin-BMCs improved cardiac functional recovery of post-MI, cardiac function was measured in the sham-control, post-MI, GFP-BMCs or apelin-BMCs treated post-MI mice. After 28 days of MI, post-MI mice had a significant lower end-systolic pressure (ESP) and +dP/dtmax pressure and a higher end-systolic volume (ESV) compared to non-ischemic sham controls (**Fig. 5 A and B**). GFP-BMCs treatment resulted in a significant decrease in ESV, but had little effects on ESP and +dP/dtmax/-dP/dtmin pressure. Treatment of post-MI mice with Apelin-BMCs significantly improved ESP and +dP/dtmax/-dP/dtmin pressure compared to GFP-BMCs treatment (**Fig. 5 A and B**).

### Knockout of Sirt3 blunts the protective effect of apelin-BMCs therapy

**Figure 6:**
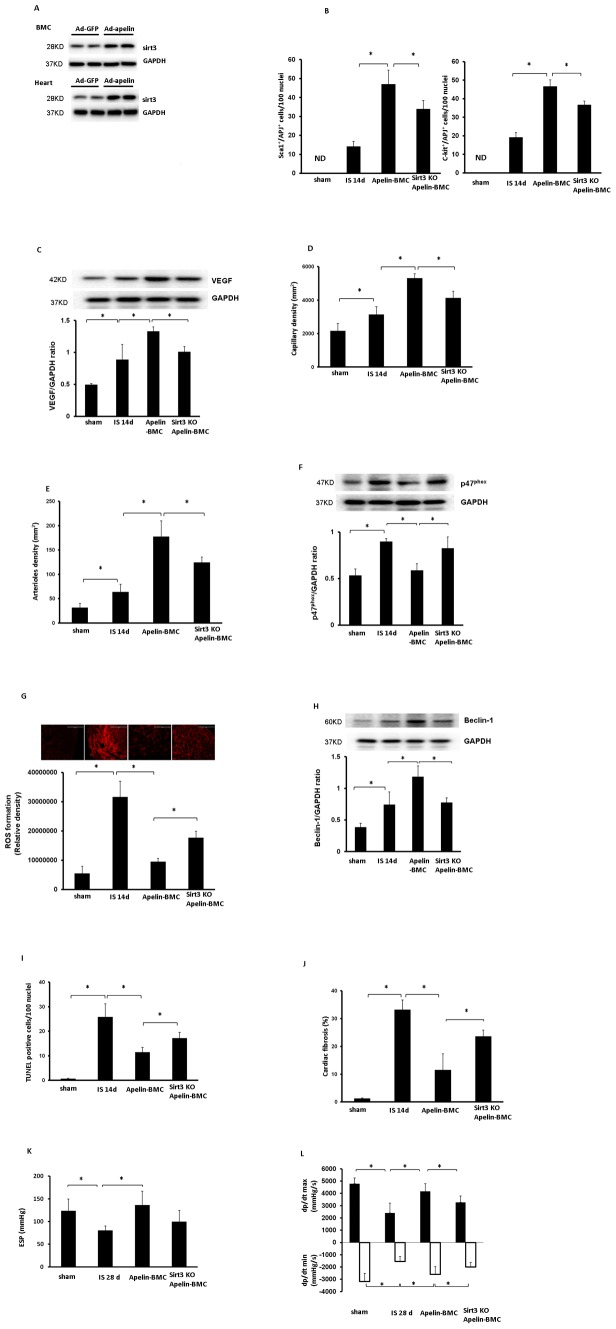
Knockout of Sirt3 blunts apelin-BMCs-mediated cardiac repair and functional recovery after MI. **A**. Systemic delivery of Ad-apelin for 5 days increased levels of Sirt3 in the BMCs and hearts. n = 2 mice. **B**. The number of Sca1^+^/APJ^+^ cells and c-kit^+^/APJ^+^ cells in the injected area was significantly reduced in post-MI mice treated with Sirt3KO-apelin-BMCs compared to apelin-BMCs treatment. n = 5, *p<0.05. ND = not detected. **C**. VEGF expression was significantly reduced in the mice treated with Sirt3KO-apelin-BMCs compared to apelin-BMCs treatment. n = 6 mice, *p<0.05. **D**. Myocardial capillary density in the border zone of ischemia area was significantly decreased in the mice treated with Sirt3KO-apelin-BMCs compared to apelin-BMCs treatment. n = 6 mice, *p<0.05. **E**. Arteriole density in the border zone of ischemia area was significantly decreased in the mice treated with Sirt3KO-apelin-BMCs compared to apelin-BMCs treatment. n = 6 mice, *p<0.05. **F**. The expression of p47^phox^ was significantly elevated in the hearts of post-MI mice treated with Sirt3KO-apelin-BMCs compared to apelin-BMCs treatment. n = 6 mice, *p<0.05. **G**. DHE staining and quantitative analysis showing that myocardial ROS formation was significantly increased in the ischemia area of post-MI mice treated with Sirt3KO-apelin-BMCs compared to apelin-BMCs treatment. n = 5 mice, *p<0.05. **H**. The expression of beclin-1 was significantly reduced in the mice treated with Sirt3KO-apelin-BMCs compared to apelin-BMCs treatment. n = 6 mice, *p<0.05. **I**. Quantitative analysis of apoptotic cells by TUNEL staining showing that TUNEL^+^ nuclei in the ischemia area was significantly increased in post-MI mice treated with Sirt3KO-apelin-BMCs compared to apelin-BMCs treatment. n = 6 mice, *p<0.05. **J**. The cardiac fibrosis was significantly increased in post-MI mice treated with Sirt3KO-apelin-BMCs compared to apelin-BMCs treatment. n = 3–5 mice, *p<0.05. **K**. Treatment with apelin-BMCs resulted in a significant increase in ESP compare to the saline-treated ischemic mice. But there were no obvious difference between Sirt3KO-apelin-BMCs treatment with saline-treated ischemic mice. n = 4–6 mice, *p<0.05. **L**. Sirt3KO-apelin-BMCs treatment significantly decreased +dP/dtmax pressure and increased –dp/dtmin pressure compared to apelin-BMCs treatment. n = 4–6 mice,*p<0.05.

Systemic delivery of Ad-apelin in donor mice for 5 days upregulated expression of Sirt3 in the hearts and BMCs, but not in the Ad-GFP treated mouse hearts and BMCs (**Fig. 6A**). To determine whether upregulation of Sirt3 contributes to apelin-BMCs mediated cardiac repair, apelin-Sirt3KO-BMCs were injected to infarcted hearts of post-MI mice. The number of APJ^+^/c-kit^+^/Sca1^+^ cells in the injected ischemic area was significantly reduced in Sirt3KO-apelin-BMCs treated mice as compared to apelin-BMCs treatment (**Fig. 6B**). Knockout of Sirt3 blunted apelin-BMCs-induced VEGF expression and angiogenesis in post-MI mice (**Fig. 6C–E**). Knockout of Sirt3 further attenuated Apelin-BMCs-mediated inhibition on p47^phox^ expression and ROS formation (**Fig. 6F&G**). Moreover, knockout of Sirt3KO in BMCs abolished apelin-induced beclin-1 expression and increased myocardial apoptosis (**Fig. 6H&I**). Myocardial fibrosis formation was also significantly increased in the apelin-sirt3KO-BMCs treated mice compared to apelin-BMCs treated mice (**Fig. 6 J**). Most intriguingly, knockout of Sirt3 diminished apelin-BMCs-mediated improvement of LV systolic function (**Fig. 6 K&L**), but had little effects on the diastolic function in post-MI mice (data not shown).

### Apelin treatment increases expression of pro-survival and pro-angiogenic growth factors in cultured BMCs

**Figure 7:**
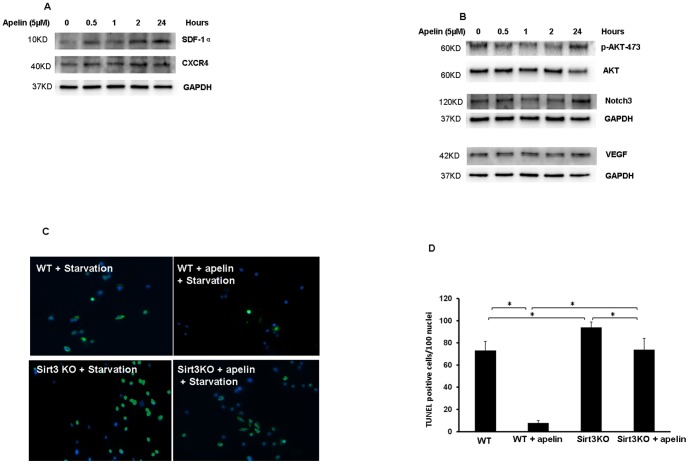
Apelin increases pro-survival and pro-angiogenic growth factors and attenuates cell apoptosis in cultured BMCs. A. Representative Western blot analysis showing that treatment of cultured BMCs with apelin (5 µM) upregulates SDF-1α and CXCR-4 expression at 0.5 hours up to 24 hours. Experiments were repeated in two cell lines. B. Representative Western blot analysis showing that treatment of cultured BMCs with apelin (5 µM) for 24 hours increases Akt phosphorylation and upregulates VEGF and Notch3 expression. Experiments were repeated in two cell lines. C and D. Representative TUNEL staining images and quantitative analysis showing that knockout of Sirt3 significantly increased starvation-induced BMCs apoptosis. Treatment with apelin resulted in a significant reduction of BMCs apoptosis whereas knockout of Sirt3 diminished apelin-mediated protective effect against cell apoptosis in cultured BMCs. n = 3, *p<0.05.

To examine the direct role of apelin on BMCs, cultured BMCs were treated with apelin (5 µM) for 0.5, 1, 2 and 24 hours, pro-survival and angiogenic growth factors were measured. Exposure of BMCs to apelin led to a gradual increase in SDF-1α and CXCR4 expression at 0.5 hours and up to 24 hours (Figure 7A). Treatment of BMCs with apelin increased expression of Notch3 and VEGF only at 24 hours. The phosphorylation of Akt-473 was also increased in apelin-treated BMCs at 24 hours (Fig. 7 B).

### Knockout of Sirt3 attenuates anti-apoptotic effect of apelin in cultured BMCs

To further confirm the histological findings and functional effect of Sirt3 observed in the apelin-BMCs treated post-MI mice, parallel studies were performed in cultured BMCs exposed to serum free medium (starvation) to induce cell apoptosis. Exposure of BMCs to apelin (5 µM) for 48 hours significantly attenuated starvation-induced apoptosis compared to BMCs without apelin treatment. Knockout of Sirt3 significantly increased starvation-induced cell apoptosis in cultured BMCs. Furthermore, knockout of Sirt3 completely abolished the anti-apoptotic effect of apelin treatment (Figure 7 C and D).

Similarly, transfection of WT-BMCs with Ad-apelin significantly attenuated starvation-induced apoptosis compared to GFP transfected BMCs. Transfection of Ad-apelin in Sirt3KO-BMCs failed to inhibit cell apoptosis (Fig. S1 A). Furthermore, transfection of WT-BMCs with Ad-apelin significantly increased BMCs proliferation as compare to GFP transfected BMCs. However, transfection of Ad-apelin in Sirt3KO-BMCs had little effect on BMCs proliferation (Fig. S1 B).

## Discussion

In the present study, we have demonstrated that treatment of post-MI mice with overexpressing apelin-BMCs upregulates expression of Sirt3 and VEGF as compare to BMCs treatment. This is accompanied by a significant increase in myocardial angiogenesis. Apelin-BMCs treatment significantly increases LC3 and beclin-1 expression and attenuates myocardial apoptosis as compare to BMCs treatment. Apelin-BMCs treatment also significantly improves cardiac function in post-MI mice. Intriguingly, knockout of Sirt3 in cultured BMCs completely abolished apelin-mediated anti-apoptotic effect. Moreover, apelin-mediated cardiac repair and functional recovery are significantly blunted in Sirt3KO-BMCs. Our study suggests that the protective mechanism of apelin-BMCs may be due, in part, to upregulation of Sirt3 signaling pathway.

Acute myocardial ischemia has been shown to induce rapid mobilization of BMCs [27], [28]. BMCs home to sites of ischemia and contribute to the neovascularization in the ischemic hearts [29], [30]. BMCs have been shown to promote cardiac repair and improve functional recovery of post-MI; however, the molecular mechanisms are incompletely understood. Previous study showed that upregulation of apelin/APJ in BMCs may facilitate improvement of myocardial functional recovery after BMCs transplantation [31]. For the first time, we showed that intramyocardial delivery of apelin-BMCs in infarcted area increased Sirt3 expression followed by a significant reduction of NADPH oxidase p47^phox^ and gp91^phox^ expression as well as ROS formation in post-MI mice. Treatment with apelin-BMCs also increased autophagy gene beclin-1 and LC3-I/II expression. These are accompanied by dramatic reduction of myocardial apoptosis. Apelin-BMCs treatment further upregulated expression of angiogenic growth factors and dramatically increased angiogenesis in the injected area. Surprisingly, no GFP^+^-BMCs or GFP^+^-apelin-BMCs were found in hearts of post-MI mice after 14 and 28 days of BMCs injection, suggesting that apelin-overexpressed BMCs had not differentiated into neovessels in ischemic hearts. These data are consistent with a recent study revealed that apelin treatment improves cardiac repair by stimulation of myocardial angiogenesis but not through BMCs transdifferentaiton [32]. Our present data further showed that treatment with apelin significantly increased stem cell recruitment factors SDF-1α and CXCR-4 expression in cultured BMCs. Injection of apelin-BMCs significantly increased the number of APJ^+^/Sca1^+^/c-kit^+^ cells at ischemic area in post-MI mice. Moreover, the number of APJ^+^/Sca1^+^/c-kit^+^ cells was significantly reduced in Sirt3KO-apelin-BMCs accompanied by a significant decline of cardiac function in post-MI mice. Taken together, these data suggested that increased number of APJ^+^ stem cell at ischemia area via upregulation of SDF-1α/CXCR-4 pathway maybe responsible for apelin-BMCs therapy-mediated cardiac repair. It is unknown whether APJ^+^ stem cell releases more proangiogenic and anti-apoptotic factors than other stem cells during ischemia. Therefore more studies are needed to investigate the functional roles of APJ^+^ stem cell on cardiac functional recovery after MI.

Our previous study indicates that apelin may promote cardiac repair and heart functional recovery of post-MI by increasing BM derived vascular progenitor cell homing and stimulating angiogenesis via a paracrine mechanism [15]. Our present data further showed that treatment with apelin significantly increased pro-survival Akt as well as angiogenic growth factor VEGF in BMCs and promoted BMC colony formation *in vitro*. Treatment of cultured BMCs with apelin or overexpression of apelin in BMCs significantly reduces stress-induced cell apoptosis. Our data also showed that treatment with apelin led to a greater phosphorylation of eNOS and upregulation of Notch3 expression in the BMCs isolated from 14 days of post-MI mice. These data suggest that overexpression of apelin in BMC may boost BMCs therapy via secretion of VEGF, activation of Notch3 and Akt/eNOS signaling pathway, which resulting in an improvement of cell survival and angiogenesis in post-MI mice. Our data further showed that injection of GFP-BMCs into ischemic hearts significantly ameliorated LV dilation, but it failed to improve LV systolic function in post-MI mice. Most intriguingly, injection of apelin-BMCs significantly improved each of these variables compared to GFP-BMCs treatment, indicating that apelin enhances cardiac systolic functional recovery of BMCs therapy in post-MI. Taken together, the present data implicate a therapeutic potential of apelin in the BMCs therapy during cardiac repair and functional recovery of post-MI.

Sirtuins are a highly conserved family of histone/protein deacetylases whose activity can prolong the lifespan of organisms [17]. Sirtuin's family (Sirtuin 1–7) has been shown to modulate distinct metabolic and stress-response pathways in mammalian cells [17], [33]. Sirtuins are involved in biological functions related to development of heart failure, including regulation of oxidative stress, angiogenesis, autophagy and apoptosis [17]–[19], [33]. To date, Sirt3 is the only gene whose increased expression was associated with extended lifespan of humans [16], [34]. Exercise and calorie restriction elevated levels of Sirt3 expression followed by improvement of cardiovascular function in aging [35]. Sirt3 also plays a critical role in the regulation of mitochondria ROS formation in aging-related cardiac hypertrophy [18], [19], [33], [36]–[38]. Our data showed that overexpression of apelin significantly upregulated Sirt3 expression and attenuated ROS formation in the heart and BMCs. In contrast, knockout of Sirt3 abolished apelin-BMCs-mediated VEGF expression and angiogenesis. Moreover, knockout of Sirt3 blunted beneficial effects of apelin-BMCs therapy. Our study also showed that knockout of Sirt3 in cultured BMCs increased stress-induced cell apoptosis and completely abolished anti-apoptotic effect of apelin treatment. These data strongly indicate that upregulation of Sirt3 in BMCs may contribute to the therapeutic benefits of apelin gene therapy in ischemic heart failure.

Autophagy has been reported to have a protective role in the heart following myocardial ischemia/reperfusion [39], [40]. In the present study, we showed that treatment of post-MI mice with apelin-BMCs increased autophagy gene LC3-I/II and beclin-1 expression together with significant reduction of myocardial apoptosis. Apelin-induced upregulation of beclin-1 expression was significantly blunted in Sirt3KO BMCs. Our data implicate that apelin-BMCs therapy may improve cardiac repair by a mechanism involving upregulation of LC3 and beclin-1 via activation of Sirt3 in ischemic hearts.

In summary, the current study provides evidence that overexpression of apelin in BMCs enhances the therapeutic efficiency of BMCs and improves systolic function in post-MI mice. Our data suggest that modification of BMCs with apelin could use as a novel cell-based therapy for the treatment of patients with myocardial ischemia and heart failure. Our findings further suggest that upregulation of Sirt3 may improve BMCs therapeutic effect for cardiac repair after MI.

## Supporting Information

Figure S1 Overexpression of apelin reduces apoptosis and increases proliferation of cultured BMCs. **A**. Representative images and quantification of serum-free (starvation) induced cell apoptosis in cultured BMCs isolated from WT and Sirt3 KO mice transfected with Ad-apelin and Ad-GFP. Transfected of BMCs with Ad-apelin attenuated starvation-induced cell apoptosis. Starvation-induced cell apoptosis was significantly increased in cultured BMCs of Sirt3KO mice transfected with Ad-apelin compared to that of WT mice transfected with Ad-apelin (n = 3 mice, *p<0.05). **B**. Transfected of BMCs with Ad-apelin significantly increased cell proliferation compared to GFP transfected BMCs. The proliferative rate of BMCs was significantly reduced in cultured BMCs of Sirt3KO mice compared to that of WT mice transfected with Ad-apelin as measured by MTT method (n = 3 mice, *p<0.05).
(DOCX)
