# Supplementary material for: Myocardial Injection of Apelin-Overexpressing Bone Marrow Cells Improves Cardiac Repair via Upregulation of Sirt3 after Myocardial Infarction
Source: PLoS One. 2013 Sep 6;8(9):e71041. doi: 10.1371/journal.pone.0071041 (PMC3765164; doi:10.1371/journal.pone.0071041)

**Supplemental Material:**

**Methods**

**Cultured BMCs proliferation and apoptosis:** Wild type (WT) mice and Sirt3 knockout mice (Jackson laboratory, Bar Harbor, ME) were sacrificed by cervical dislocation under anesthesia with isoflurane. BM–derived mononuclear cells were obtained by flushing the tibias and femurs with 10% FBS DMEM. Immediately after isolation, 10^5^ BM–derived mononuclear cells were plated into 6-well culture plates. After 4 days of culture, the nonadherent cells were removed and the adherent cells were washed three times with phosphate-buffered saline solution (PBS) ^1, 2^. Deficiency of Sirt3 in the bone marrow cells was verified by western blot analysis. BMCs were then infected with Ad-GFP (10^8^ PFU) or Ad-apelin (10^8^ PFU) in culture medium.

For the cell proliferation measurement, BMCs were cultured in 10%FBS DMEM for 72 hours. The proliferative capacity of cultured BMCs was assayed using a cell proliferation (MTT) kit according to the manufacturer's instructions (Roche Diagnostic Corp., IN, USA)^3, 4^. In the apoptosis study, BMCs apoptosis was induced by exposure of cultured BMCs to serum-free medium for 48 hours. The number of apoptotic cells was then examined by counting TUNEL positive cells per 100 nuclei in cultured BMCs.

**FIGURE LEGENDS**

**Figure S 1. Overexpression of apelin reduces apoptosis and increases proliferation of cultured BMCs**

**A.** Representative images and quantification of serum-free (starvation) induced cell apoptosis in cultured BMCs isolated from WT and Sirt3 KO mice transfected with Ad-apelin and Ad-GFP. Transfected of BMCs with Ad-apelin attenuated starvation-induced cell apoptosis. Starvation-induced cell apoptosis was significantly increased in cultured BMCs of Sirt3KO mice transfected with Ad-apelin compared to that of WT mice transfected with Ad-apelin (n=3 mice, *p<0.05).

**B.** Transfected of BMCs with Ad-apelin significantly increased cell proliferation compared to GFP transfected BMCs. The proliferative rate of BMCs was significantly reduced in cultured BMCs of Sirt3KO mice compared to that of WT mice transfected with Ad-apelin as measured by MTT method (n=3 mice, *p<0.05).

Reference List

(1) Tuo QH, Zeng H, Stinnett A et al. Critical role of angiopoietins/Tie-2 in hyperglycemic exacerbation of myocardial infarction and impaired angiogenesis. *Am J Physiol Heart Circ Physiol* 2008 June;294(6):H2547-H2557.

(2) Li L, Zeng H, Chen JX. Apelin-13 increases myocardial progenitor cells and improves repair postmyocardial infarction. *Am J Physiol Heart Circ Physiol* 2012 September 1;303(5):H605-H618.

(3) Chen JX, Stinnett A. Critical role of the NADPH oxidase subunit p47(phox) on vascular TLR expression and neointimal lesion formation in high-fat diet-induced obesity. *Lab Invest* 2008 September 8.

(4) Chen JX, Tuo Q, Liao DF, Zeng H. Inhibition of protein tyrosine phosphatase improves angiogenesis via enhancing Ang-1/Tie-2 signaling in diabetes. *Exp Diabetes Res* 2012;2012:836759.

Fig S1 A


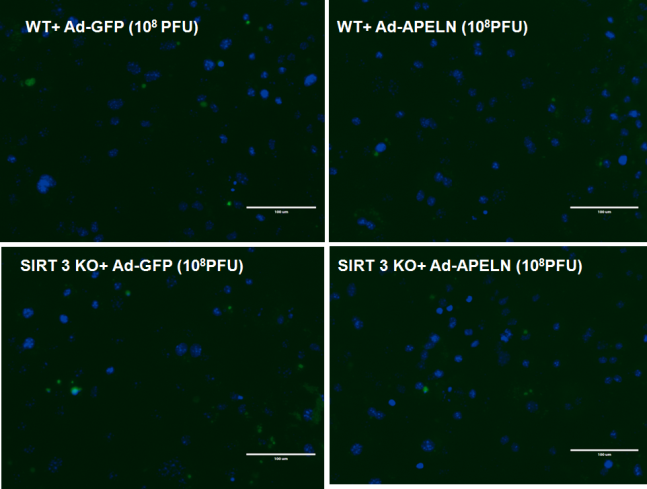

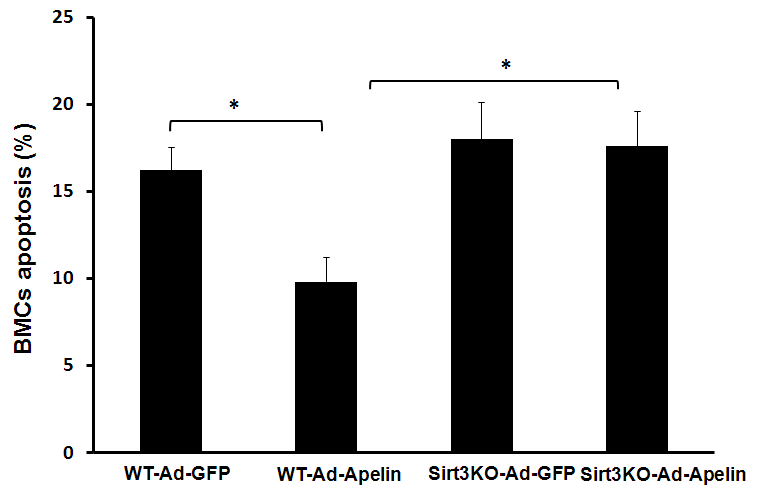


Fig S1B


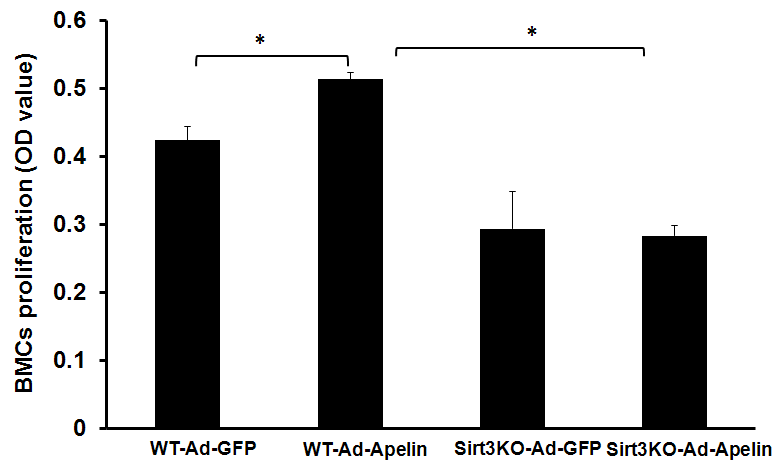

Supplement: Figure S1 — Overexpression of apelin reduces apoptosis and increases proliferation of cultured BMCs. A. Representative images and quantification of serum-free (starvation) induced cell apoptosis in cultured BMCs isolated from WT and Sirt3 KO mice transfected with Ad-apelin and Ad-GFP. Transfected of BMCs with Ad-apelin attenuated starvation-induced cell apoptosis. Starvation-induced cell apoptosis was significantly increased in cultured BMCs of Sirt3KO mice transfected with Ad-apelin compared to that of WT mice transfected with Ad-apelin (n = 3 mice, *p<0.05). B. Transfected of BMCs with Ad-apelin significantly increased cell proliferation compared to GFP transfected BMCs. The proliferative rate of BMCs was significantly reduced in cultured BMCs of Sirt3KO mice compared to that of WT mice transfected with Ad-apelin as measured by MTT method (n = 3 mice, *p<0.05). (DOCX) [file pone.0071041.s001.docx]
